# A Population Pharmacokinetic Approach to Describe Cephalexin Disposition in Adult and Aged Dogs

**DOI:** 10.1155/2014/789353

**Published:** 2014-11-06

**Authors:** Ana Paula Prados, Paula Schaiquevich, Verónica Kreil, Agustina Monfrinotti, Pamela Quaine, Lisa Tarragona, Ruben Hallu, Marcela Rebuelto

**Affiliations:** ^1^Farmacología, Facultad de Ciencias Veterinarias, Universidad de Buenos Aires. Chorroarín 280, 1427 Buenos Aires, Argentina; ^2^Unidad de Farmacocinética Clínica-CONICET, Hospital de Pediatría JP Garrahan, Combate de los Pozos 1881, 1245 Buenos Aires, Argentina

## Abstract

This study was conducted in order to characterize the pharmacokinetics of orally administered cephalexin to healthy adult and aged dogs, using a population pharmacokinetic approach. Two hundred and eighty-six cephalexin plasma concentrations obtained from previous pharmacokinetic studies were used. Sex, age, pharmaceutical formulation, and breed were evaluated as covariates. A one-compartment model with an absorption lag-time (Tlag) best described the data. The final model included age (adult; aged) on apparent volume of distribution (Vd/F), apparent elimination rate (ke/F), and Tlag; sex (female; male) on ke/F, and breed (Beagle; mixed-breed) on Vd/F. Addition of the covariates to the model explained 78% of the interindividal variability (IIV) in Vd/F, 36% in ke/F, and 24% in Tlag, respectively. Formulation did not affect the variability of any of the pharmacokinetic parameters. Tlag was longer, whereas Vd/F and ke/F were lower in aged compared to adult animals; in female aged dogs ke/F was lower than in male aged dogs; however, the differences were of low magnitude. Different disposition of cephalexin may be expected in aged dogs.

## 1. Introduction

Cephalexin is a beta-lactam antibiotic frequently used in veterinary practice. Due to its bactericidal activity against common pathogens and lack of toxicity, cephalexin is recommended for treating various skin and soft tissue infections in dogs of all ages when gram-positive cocci (i.e., staphylococci) are the causative agents. The time-dependent bactericidal activity of cephalexin is closely related to its plasma concentrations, which must remain over the minimum inhibitory concentration (MIC) of the offending pathogen for at least 50% of the dosing interval [1, 2]. Thus, any change in cephalexin plasma concentrations may result in an altered response to the therapeutic intervention.

Age has been previously described as potential source of variability in the pharmacokinetics of drugs, translating into changes in the pharmacological response. Several physiological processes involved in the absorption (gastrointestinal motility, gastric acid secretion), distribution (blood flow distribution, total body water, and protein plasma binding), and elimination (hepatic and renal functions) of drugs may be impaired in geriatric human and veterinary patients [3–6]. Thus, concerns arise regarding cephalexin absorption and disposition when administered to geriatric dogs. However, despite the extensive clinical use of this antibiotic in aged dogs, reports of its pharmacokinetic behaviour in this population are lacking. This may be due to the difficulty in obtaining sufficient data to perform a conventional pharmacokinetic study.

The pharmacokinetics of cephalexin following its oral administration to dogs has been previously described using the conventional noncompartmental analysis in different situations that may affect cephalexin disposition, requiring dose adjustment. The lack of effect of food on the absorption of cephalexin in the dog has been reported by Campbell and Rosin [7] and Chicoine et al. [8]. Previous administration of metoclopramide significantly increased cephalexin peak plasma concentration and area under the curve values [9], whereas meloxicam did not affect cephalexin pharmacokinetics [10]. A chronokinetic study concluded that cephalexin pharmacokinetics vary with time of day administration [11]. Carli et al. [12] described the compartmental pharmacokinetic analysis of intravenous, intramuscular, and oral cephalexin and calculated a moderate oral bioavailability (57 ± 5%) of this drug in dogs.

Currently, there is interest in gathering pharmacokinetic data to enable a new approach to the rational design of dosage regimens by means of the population pharmacokinetic analysis [5, 13]. This pharmacokinetic study may be appropriate to identify the presence of subpopulations by means of a covariate analysis. Thus, this study was conducted in order to characterize the pharmacokinetics of orally administered cephalexin to healthy adult and aged dogs, using a population pharmacokinetic approach.

## 2. Materials and Methods

### 2.1. Study Data

Data for the present analysis were obtained from previous studies [9–11] and unpublished data conducted in order to characterize the pharmacokinetics of cephalexin in dogs. Animals were obtained from the kennels of Facultad de Ciencias Veterinarias, Universidad de Buenos Aires, and none of them had a history of allergy to beta-lactams. The characteristics of the studied population are represented in Table 1. The dogs were housed in a controlled environment during the entire experience. Protocols were approved by the Institutional Animal Care and Use Committee of the Veterinary Science School, University of Buenos Aires (protocol number 2010/20).

### 2.2. Drug Administration

The experimental design was similar in all studies. In brief, dogs received a single oral 25 mg/kg dose of cephalexin in the morning (8.00-9.00 a.m.), after an overnight fast. Food and water were given 6 and 2 h after drug administration, respectively. All the animals were weighed before each treatment. Cephalexin monohydrate as a 5% aqueous solution was administered in 14 occasions (Cefalexina 250 mg, Bio-Amer, Buenos Aires, Argentina) and cephalexin 500 mg tablet (Cefalexina 500, Holliday, Buenos Aires, Argentina) was administered in 8 occasions to the same animals after a wash-out period of not less than a week. Animals were randomized to the sequence of the pharmaceutical form. Blood samples (2 mL) were drawn from the jugular vein into heparinized tubes at the following times: 0, 0.16, 0.33, 0.5, 0.75, 1, 1.5, 2, 2.5, 3.5, 5.5, 7.5, 9.5, and 11.5 h after cephalexin administration. Samples were centrifuged at 3,000 rpm for 15 min and plasma was collected and kept frozen at −20°C until cephalexin quantitation by a validated microbiological assay [9–11]. Further details of the pharmacokinetic studies including bioanalytical methods have been described elsewhere [9–11].

### 2.3. Population Pharmacokinetic Analysis

Data were analyzed using the nonlinear mixed effect modelling software program Monolix version 4.2 (Lixoft, Orsay, France). Pharmacokinetic parameters were estimated using the stochastic approximation expectation maximization algorithm (SAEM) combined with a Markov chain Monte Carlo procedure (MCMC). The number of MCMC chains was fixed to 50 for all estimations.

One- and two-compartment models with and without lag-time were evaluated with different residual errors to identify the model that best described the set of data. Interindividual variability (IIV, i.e., the variability among the dogs) and interoccasion variability (IOV, i.e., variability between the two pharmacokinetic studies) were modelled using an exponential model. Proportional, additive, and combined (additive and proportional) error models were evaluated to describe the residual variability.

The likelihood ratio test was used to test different models for modelling the residual variability and the covariate effect on each pharmacokinetic parameter. A univariate analysis of each covariate in all pharmacokinetic parameter was carried out to evaluate the significance on the model and was retained if the objective function value was reduced by at least 3.84 units (*P* < 0.05, one degree of freedom). Afterwards, the forward stepwise approach followed by a backwards approximation was implemented to define the final model. In addition, diagnostic plots, the distribution of errors, and the precision of the parameter estimates were used as tools to evaluate the goodness of fit and to compare between models.

Sex, age, type of the administered pharmaceutical formulation, and breed were evaluated as covariates to study whether they explained a significant portion of the interindividual variability of each pharmacokinetic parameter. Covariates were evaluated as categorical (0 or 1). Then, the effect of age was set as 0 = adult; 1 = aged dog; breed, 0 = mixed breed and 1 = Beagle; pharmaceutical formulation, 0 = solution and 1 = tablet; and sex, 0 = female and 1 = male.

In order to evaluate the performance of the final model, a visual predictive check was carried out based on the parameters and random effects obtained in the final model and using those values to simulate a thousand datasets. The 90% prediction intervals were constructed and plotted together with the observed data so as to assess visually the agreement between simulations and observations.

## 3. Results

A total of 14 dogs were evaluated in 22 pharmacokinetic studies. A total of 286 plasma concentrations were available for pharmacokinetic analysis. A one-compartment model with an absorption lag-time best described the data. Thus, pharmacokinetic models were parameterized in terms of absorption rate constant (ka), apparent first order elimination rate constant (ke/F), lag-time (Tlag), and apparent volume of distribution (Vd/F). Interindividual variability and interoccasion variability were incorporated in all four parameters. Residual variability was described by a combination error model. A total of 55 concentrations were below the limit of quantitation (0.78 *μ*g/mL) and thus were modelled as censored data.

Covariates were evaluated in all four pharmacokinetic parameters in order to check whether there was a significant decrease in the objective function and the interindividual variability of each parameter.

Table 2 summarizes some of the model building steps showing only those covariates that were significantly related to the pharmacokinetic parameters.

As shown in Table 3, the final population pharmacokinetic estimates were well estimated. By adding age on Vd/F, ke/F, and Tlag, sex on ke/F, and breed on Vd/F, we could explain 78% of the interindividual variability (IIV) in Vd/F, 36% of the IIV in ke/F, and 24% of the IIV in Tlag, respectively. Based on the pharmacokinetic model described in Table 3, we proposed a mean (s.e) Tlag for aged and adult dogs of 0.59 h (0.25) and 0.14 h (0.05), respectively. In addition, adult dogs mixed breed and pure breed means (s.e) Vd/F were 565 mL/kg (30) and 791 mL/kg (42), respectively. On the other hand, for aged dogs with mixed breed and pure breed, the means (s.e) Vd/F were 440 mL/kg (28) and 616 mL/kg (36), respectively. Finally, female aged dogs' mean (s.e) kel/F was 0.291 h^−1^  (0.015) while, for males, the mean value of the parameter was 0.330 h^−1^  (0.018).

**Figure 1:**
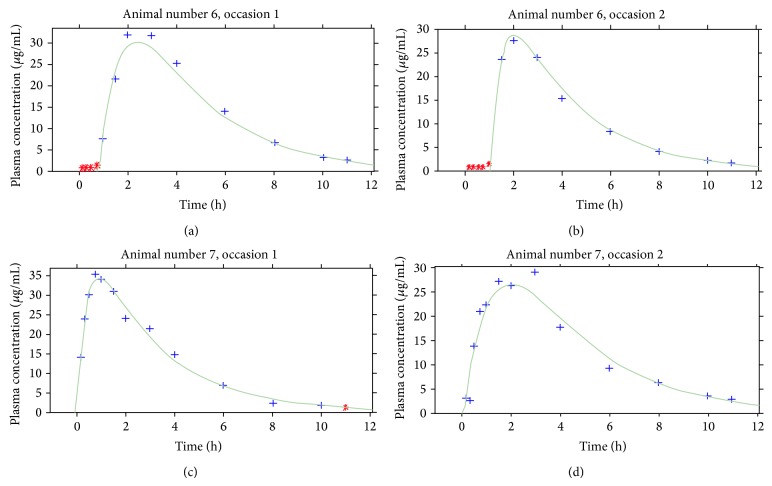
Concentration versus time profiles of cephalexin. Symbols represent the observed data for two representative animals studied in two occasions: green line, individual estimation.

**Figure 2:**
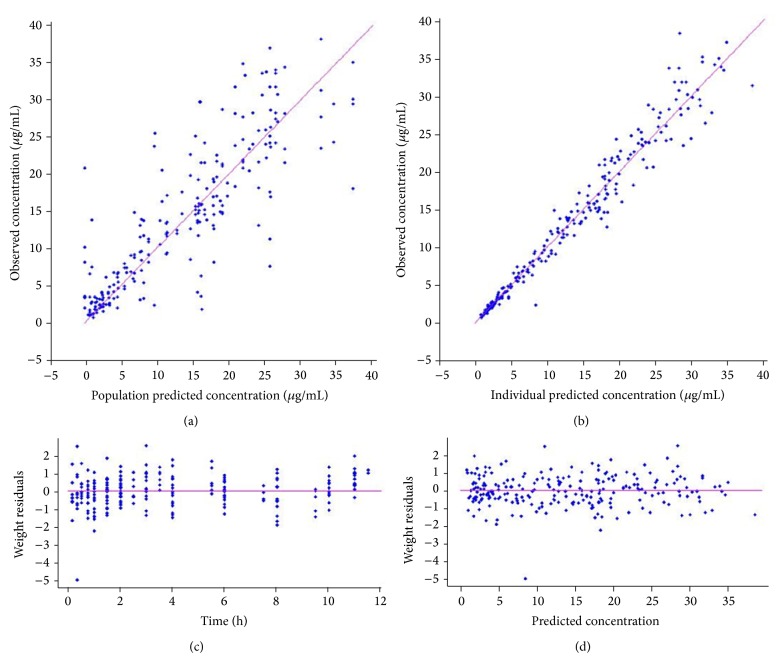
Goodness of fit plots. (a) Observed versus population predicted and (b) observed versus individual predicted values. The continuous line is the identity line, (c) scatter plot of weighted residuals versus time and (d) scatter plot of weighted residuals versus predicted cephalexin concentrations.

**Figure 3:**
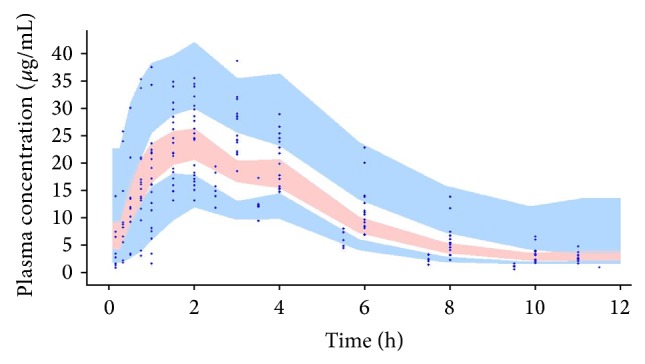
Visual predictive check. The figure shows the 90% prediction intervals obtained by simulation using the final model. The light blue areas are the 90% confidence intervals for the median, 5th percentile, and the 90th percentile of the simulated data. Circles represent observed data.

For two representative animals in two different occasions (receiving cephalexin solution in occasion 1 and tablet in occasion 2), observed concentration versus time profiles along with the individual and population estimates are depicted in Figure 1. In addition, the goodness-of-fit plots are depicted in Figures 2(a) and 2(b). Observed versus model and individual predicted concentrations were spread around the line of identity. Besides, no trend in the residuals plots over time or with respect to the observed concentration could be observed (Figures 2(c) and 2(d)). Overall, the visual predictive check showed no bias in the model prediction with respect to the observed data and most of the observed data were within the 90% prediction interval (Figure 3).

## 4. Discussion

Antibiotic optimal dosage regimens are aimed at warranting clinical efficacy and avoiding or minimizing the selection of resistant microorganisms. Accurate pharmacokinetic and pharmacodynamic data are the basis for a rational antimicrobial therapeutic regimen. Beta-lactam antibiotics, including cephalexin, are frequently used in the clinical practice of aged animals due to their low toxicity and wide antibacterial activity. However, pharmacokinetic studies in the geriatric population are scarce.

This study was conducted in order to develop a population pharmacokinetic model, using data of previous studies in our laboratory describing the pharmacokinetics of cephalexin following its oral administration in two different formulations to healthy dogs [9–11]. Different demographic, clinical, and biochemical characteristics referred to as covariates in population pharmacokinetics may explain large percentage of the interindividual variability in the pharmacokinetic parameters. Thus, knowing those sources of variability allows for individualization of the dose according to the characteristics of each patient. Age, sex, and breed are the most frequently registered covariates in the clinical veterinary practice; thus, those were selected as individual characteristics in this analysis.

A one-compartment open pharmacokinetic model with first order absorption with an absorption lag-time best fits the plasma concentration-time data. In this final population model, the interindividual variability in Tlag, Vd/F, and ke/F could partially be explained by age; breed and age; and sex and age, respectively, whereas the other covariate tested (formulation) did not affect the variability of any of the pharmacokinetic parameters.

Drug absorption processes are especially complex when the oral route is used, as the drug may traverse the gut wall by diverse mechanisms, that is, passive diffusion and carrier-mediated transport. In addition, drugs may further suffer extrusion back into the intestinal lumen by members of the ATP binding cassette transporter family (i.e., P-glycoprotein, multidrug resistance-associated protein 2, MRP2, and breast cancer resistance protein, BCRP) localized in the apical membrane of the enterocyte [14]. It has been previously determined that cephalexin is a substrate of the intestinal dipeptide transporter PEPT1 [15, 16]. Moreover, cephalexin intestinal absorption is primarily mediated by PEPT1 in rats and mice [17, 18]. Our results showed that age modifies the time to cephalexin appearance in the systemic circulation, as Tlag was higher in geriatric animals. However, absorption rate was not modified by age. Small increases in gastrointestinal transit times have been found in aged animals [3, 5, 6] and may account for this difference, as lag-time reflects not only the release from the formulation but also the drug migration to the absorbing surface [19]. Similarly, in a pharmacokinetic study of orally administered amoxicillin to dogs, a high Tlag value was determined in one geriatric dog. However, Tlag was substantially reduced when amoxicillin was coadministered with metoclopramide, a prokinetic agent, in the same dog [20].

Age and breed of dogs were important covariables in the variability of the median parameter Vd/F in dogs. Our study showed that cephalexin Vd/F is lower in the mixed-breed geriatric dogs and higher in the Beagle adult dogs; however, all values were congruent with the reported range of total body water (0.556–0.660 L/kg) in healthy nonobese dogs [21]. This was expected due to the polar nature of first-generation cephalosporins. Several factors affect distribution of drugs, including body composition, regional blood flows, and plasma protein binding. A breed effect on body composition [22, 23] and on plasma lipoprotein lipids [24] has been described in dogs. In addition, in geriatric dogs, changes such as decreased serum albumin, increased adipose tissue, decreased muscle mass, decreased total body water, and blood flow redistribution may be expected [3, 5, 6]. These factors may account for the difference found in our study; however, it has been determined that cephalexin protein plasma binding is low in dogs [25].

Age and sex of dogs were important covariates in Ke/F of these experimental dogs. Using our final model estimates of Ke/F, cephalexin half-life ranged from 1.8 h (adult male dogs) to 2.38 h (aged female dogs). These values are similar to those calculated by standard analysis previously reported by Campbell and Rosin [7] for fasted (1.8 h) and fed (2.6 h) dogs; Carli et al. [12] (149.5 min), Prados et al. [11] in active (1.8 h) and resting (2.7 h) phases; Prados et al. [9] (1.8 h) and [10] (2.26 h), and Chicoine et al. [8] for fasted (2.99 h) and fed (2.96 h) dogs.Cephalexin is primarily excreted by the kidney by both glomerular filtration and active OAT-mediated excretion. The decreased renal function observed in aged animals, due to the decline in the renal blood flow, glomerular filtration rate, and loss of functional nephrons [3, 5, 6], may account for the slower elimination of cephalexin in aged dogs. In our study, sex significantly influenced the variability of ke/F. It has to be emphasized that our female and male dogs were not castrated. Sex has also been previously identified as a significant covariate in the distribution of propofol in dogs [26]. In rats and mice, sex-hormone regulated differences in the function of transporters of specific organic compounds in the apical and basolateral membrane of nephron epithelial cell have been reported [27–29]. However, data obtained from experiments in rats and mice cannot be extrapolated to other species [5, 30]. The sex-related differences in drug's pharmacokinetics clinical relevance in veterinary medicine may be low [5]. In addition, veterinary patients, that is, cats and dogs, are seasonal breeders and are usually castrated when young [5].

This study has some limitations that should be accounted for. First, it was conducted in healthy animals, not in the intended target clinical population. Secondly, a small number of aged dogs were included, due to practical and ethical reasons. However, several studies on geriatric population have been conducted in healthy animals, providing results of clinical relevance, and our population pharmacokinetic approach was able to identify differences in cephalexin relevant pharmacokinetic parameters partially explained by aging.

## 5. Conclusions and Clinical Relevance

Oral absorption of cephalexin is mediated by dipeptide transporters and diffusion mechanisms, whereas elimination is mediated primarily by the kidney. Therefore, age-related impaired gastrointestinal or renal functions may lead to low plasma concentrations and, eventually, to treatment failure or development of resistant bacterial strains. In this study we have demonstrated, using a population pharmacokinetic approach, that different absorption and disposition of cephalexin may be expected in aged dogs. The pharmacokinetic parameters differences are of low magnitude, and cephalexin exhibits a wide therapeutic index; thus a dose adjustment may not be needed. However, identification of age as the covariate with frequent influence on cephalexin pharmacokinetic parameters supports further clinical studies in aged dogs.

## Figures and Tables

**Table 1:** Characteristics and treatment of the animals.

Characteristic	
Breed	Beagle = 9; mixed breed = 5

Age (median, range)	Aged (*n* = 5); 12 years (10–13)
	Adult (*n* = 9); 3 years (2–8)

Sex	Female = 7; male = 7

Formulation (number of pharmacokinetic studies)	Solution (14); tablet (8)

Dose schedule	25 mg/kg

Body weight (median, range)	Adults 12 kg (8–26)
	Aged dogs 14 kg (10–24)

**Table 2:** Pharmacokinetic model building.

Model	Change in objective function	Percentage of the interindividual variability of the pharmacokinetic parameter (%)
Univariate analysis		

Effect of age on Tlag Tlag = Tlag_typical_ × exp⁡(Age)	−5	25.2

Effect of age on Vd/F Vd/F = Vd/F_typical_ × exp⁡(Age)	−5	20.7

Effect of age on ke/F Ke/F = Ke_typical_ × exp⁡(Age)	−6	23.8

Effect of breed on Vd/F Vd/F = Vd/F × exp⁡(breed)	−9	38.3

Effect of sex on ke/F Ke/F = Ke_typical_ × exp⁡(SEX)	−5	19

Tlag: lag-time; ke/F, apparent elimination rate constant; Vd/F, apparent volume of distribution.

References: effect of age: 0 = adult; 1 = aged dog; effect of breed: 0 = mixed breed, 1 = Beagle; effect of sex: 0 = female, 1 = male. For all models, the residual variability was modelled as a combination error model.

**Table 3:** Parameter estimates of the base and the final population pharmacokinetic model for cephalexin.

Parameter	Base model mean estimate (s.e)	Final model mean estimate^a^ (s.e)
Tlag (h)	0.245 (0.082)	0.143 (0.051)
Ka (h^−1^)	1.40 (0.15)	1.39 (0.15)
Vd/F (mL/kg)	642 (44)	565 (30)
Ke/F (h^−1^)	0.341 (0.014)	0.340 (0.014)
Residual variability		
Proportional (%)	0.131 (0.009)	0.133 (0.009)
Additive (mg/L)	0.112 (0.036)	0.100 (0.03)

Tlag: lag-time; ke/F, apparent elimination rate constant; Vd/F, apparent volume of distribution.

^a^The final model corresponds to Tlag (h) = 0.144 ×exp⁡(1.42 × age); Vd/F (mL/kg) = 565 ×exp⁡(−0.25 × age)×exp⁡(0.337 × breed); Ke (h^−1^) = 0.34 ×exp⁡(0.124 × sex)×exp⁡(−0.154 × age).
